# Treatment of Plantar Fasciitis in Patients with Calcaneal Spurs: Radiofrequency Thermal Ablation or Extracorporeal Shock Wave Therapy?

**DOI:** 10.3390/jcm12206503

**Published:** 2023-10-13

**Authors:** Nevsun Pihtili Tas, Oğuz Kaya

**Affiliations:** 1 Department of Physical Medicine and Rehabilitation, Health Sciences University Elazig Fethi Sekin City Hospital, Elazıg 23280, Turkey; 2 Department of Orthopedics and Traumatology, Elazig Fethi Sekin City Hospital, Elazıg 23280, Turkey; oguzkayamd@gmail.com

**Keywords:** heel spur, extracorporeal shockwave therapy, radiofrequency ablation, visual analog scale, foot function index, the Roles and Maudsley score

## Abstract

Background and Objectives: We aimed to compare the effectiveness of ESWT (Extracorporeal Shock Wave Therapy) and RFA (Radiofrequency Thermal Ablation) on pain, disability, and activity limitation in the treatment of plantar fasciitis in patients with calcaneal spurs. Materials and Methods: Patients who apply to Orthopedics and Traumatology and Physical Medicine and Rehabilitation departments with a complaint of heel pain are included in this retrospective study. We included patients diagnosed with calcaneal spurs who received treatment with ESWT (*n* = 80) and RFA (*n* = 79) between 1 August 2021 and 1 September 2022. All patients were evaluated using the Visual Analog Scale (VAS), Foot Function Index (FFI), and the Roles and Maudsley score (RM) before and after treatment. An evaluation was performed on average 6 months after treatment. Results: This study included 79 RFA patients (34 females and 45 males) with a mean age of 55.8 ± 9.6 years and 80 ESWT patients (20 females and 60 males) with a mean age of 49.1 ± 9.5 years. There was a significant decrease in VAS scores after treatment in both the RFA and ESWT groups (z: −4.98, z: −5.18, respectively, *p* < 0.001). The reductions in FFI pain, FFI activity restriction, FFI disability, and RM scores were significant in both groups, although the scores after treatment were lower in the RFA group. Conclusions: This study demonstrates that ESWT and RFA significantly reduced pain, disability, and activity restriction in the treatment of plantar fasciitis in patients with calcaneal spurs. ESWT proved particularly effective in alleviating pain, whereas RFA had more pronounced effects on reducing disability and activity limitations. The choice of treatment should be based on the patient’s specific complaints.

## 1. Introduction

There are many causes of heel pain. Heel pain occurs as a result of systemic diseases or as a result of diseases that concern the bone, soft tissue, and nervous system. Among the causes of heel pain are tendonitis, bursitis, tarsal tunnel syndrome, neuroma, peripheral nervous system diseases, fractures, benign and malignant tumors of the bone, bone cysts, osteomyelitis, and systemic and rheumatological diseases [1,2]. Plantar fasciitis (PF) is the most common cause of heel pain [3]. High rates of calcaneal spurs (CSs) have been detected in patients with PF [4]. Right in front of the medial of the calcaneal tuberculin are fibro-cartilaginous triangular protrusions of different sizes [5]. This condition affects 15–20% of the general population [5]. Pain can affect adults of all ages, regardless of an active or sedentary lifestyle [6]. However, as the age increases, the incidence of a CS increases due to the shortening of the length of the step and the contact of the middle foot and heel to more relative places [7].

Many factors are proposed in the etiology of CSs. These factors include age, sex, obesity, excessive exercise, foot structure and biomechanics, foot injuries, rheumatoid arthritis, diabetes, osteoarthritis, and gout, which can also be considered as contributing to plantar fasciitis [8]. A physical exam and the patient’s medical history are used to make the diagnosis. In some cases, there may be no clinical evidence [9]. The classic symptom is moderate to high heel pain. Pain is felt when awakened from sleep. Reduces as you walk. The medial calcaneus in examination is sensitive and painful with palpation. Some patients experience pain during ankle dorsiflexion [10]. Direct graphs support diagnosis with symptoms and examination [11]. Although magnetic resonance imaging (MRI) and ultrasonography (US) have advantages in directly evaluating plantar fascia, radiographs allow the evaluation of conditions such as tumors, fractures, and calcaneal spurs [12,13].

Conservative methods are the first step of treatment. Rest, soft shoes, insoles, exercises, and non-steroidal anti-inflammatory drugs are conservative methods used for treatment. In patients who do not respond to conservative treatment, methods like steroid or PRP (platelet-rich plasma) injections, physical therapy modalities, ESWT (Extracorporeal Shock Wave Therapy), and RFA (Radiofrequency Thermal Ablation) are commonly used. In cases resistant to conservative and minimally invasive methods, surgical methods can be used. Surgical treatment can lead to complications and is not a definitive method of treatment [2,14,15]. The main goal of treatment is to reduce pain and improve quality of life.

Extracorporeal shock wave therapy (ESWT), which has been shown to be an effective and safe treatment for CS, has been widely used recently [16]. ESWT has been found to significantly reduce pain in patients with symptomatic calcaneal spurs [17]. ESWT is also used for shock waves, painful symptoms, and calcific accumulation. Low-energy ESWT is used in the treatment of enthesopathy and local painful conditions of the musculoskeletal system [18,19]. ESWT is a non-invasive method that stimulates micro-vascularization by creating controlled microtrauma through sound waves to the tissue. It also induces the release of enzymes that affect nociceptors and provides localized analgesia [17]. ESWT has been shown to be effective in reducing PF pain due to CSs [20].

Radiofrequency ablation (RFA) is a symptomatic, pain-relieving treatment option. RFA administration is used in the management of many painful conditions that require ablation of different nerve settlements, such as trigeminal neuralgia, complex regional pain syndrome, chronic postoperative pain, cancer pain, hyperhidrosis, facet joint pain, and knee osteoarthritis [21,22]. It is considered a safe and effective treatment for these types of heel pain [21,22]. In this procedure, a constant high-frequency, high-temperature electric current is applied to the target tissue [23].

In our study, we compared the effects of RFA and ESWT methods, of plantar fasciitis in patients with calcaneal spurs on pain and quality of life. In our literature review, we did not come across any other studies comparing the effectiveness of ESWT and RFA in the treatment of plantar fasciitis in patients with calcaneal spurs

## 2. Materials and Methods

This retrospective study was conducted on patients who complained of heel pain, received ESWT treatment at the Physical Medicine and Rehabilitation Clinic (FTR), and received RFA treatment at the Orthopedics and Traumatology clinic between August 2021 and September 2022. As a result of clinical examination and direct X-ray films patients with calcaneal spur were included. Patients who have not benefited from conservative treatments involving oral anti-inflammatory medication, insoles, and corticosteroid injection have not been treated for any medical treatment, injection, physical therapy, or surgical treatment, and RFA and ESWT have been selected for the past four weeks. Patients for whom pre-and/or post-treatment records, direct X-ray radiographic images, and historical data could not be fully obtained were excluded from this study. Data from 395 patients were evaluated, and 236 patients were not included in this study. This study consisted of 79 patients from the RFA group and 80 patients from the ESWT group. This study was agreed to by the Fırat University Ethics Committee.

There were two groups of patients. The ESWT group was selected from patients who were presented to the FTR clinic with plantar fasciitis pain due to calcaneal spurs, did not respond to conservative treatment, and did not receive RFA. The first group was treated with ESWT (Chattanooga Intelect^®^ RPW 2 (DJO LLC CA, Vista, CA, USA) in the physiotherapy unit, with 3 weekly sessions repeated over 3 weeks, each consisting of a maximum of 2000 pulses at a frequency of 10 Hz. ESWT pressure was adjusted according to the patient’s pain resistance. The applicator was placed over the maximum sensitivity point. Local or regional anesthesia was not applied. The treatment was performed with a cold gel. The second group was selected from patients who attended the Orthopedics and Traumatology clinic with plantar fasciitis pain due to calcaneal spurs, did not respond to conservative treatment, and did not undergo ESWT. The second group was treated with radiofrequency ablation (RFA). After the heel area was sterilized in the patient supine position, pain localization was detected by palpation, and local anesthesia was provided to the heel medial site with 1 mL %2 Lidocaine. In the desired location, a 10 mm needle cannula was inserted. The needle of the cannula was removed, and a 100 mm long, 22-gauge RFA (radiofrequency ablation) needle (JK2, NeuroTherm, Wilmington, MA, USA) was inserted into the cannula. First, patients were provided with a 1.5 V stimulus, and it was observed that there was no motor stimulation, such as fasciculation or movement of the toes. It was confirmed that the impedance was between 300 and 500. Thermal RFA was applied for 120 s at 60–80 °C, with the temperature increasing up to 80 °C. Thermal RFA was applied for a total of 180 s, with an additional 60 s while monitoring the impedance. In patients who reported severe pain at 80 °C, the temperature was lowered to 60 °C. Considering that patients may experience mild pain (at a tolerable level for the patient) if they do not feel any pain below 60 °C, the procedure was continued between 60 °C and 80 °C. Thus, the effectiveness of the procedure was monitored in communication with the patient. RFA is applied to patients who have not been relieved by conservative or steroid treatment. An evaluation was performed on average 6 months after the treatment.

In this study, Visual Analog Scale (VAS) scores with a range of 100 mm were used to assess pain intensity, and Foot Function Index (FFI) and Roles and Maudsley score (RM) scores were used to assess foot pain, disability, and activity limitation.

Visual Analog Scale (VAS): A table used for digitizing values that cannot be measured numerically. The patient’s condition is marked in a 100 mm line. It is a common, reliable test and can be applied easily.

Foot Function Index (FFI): Created to assess the impact of foot pathology on function in terms of pain, disability, and activity limitation. FFI is an index of 23 items divided into 3 subscales. The FFI has been reviewed for test reliability [24].

The Roles and Maudsley score (RM): A subjective evaluation of the patient’s pain and activity limitations: (1 = excellent result with no symptoms after treatment; 2 = significant improvement compared to before treatment; 3 = some improvement in the patient; and 4 = weak, the symptoms are the same as or worse than before treatment) [25].

No complications (soft tissue infection, symptomatic hematoma, nerve damage, or plantar fascia rupture) were observed with RFA and ESWT.

This study was approved by the local ethics committee (decision date: 27 May 2021; decision number: 10 July 2021).

The accession numbers will be provided during the review.

### The Statistical Analysis

The data were analyzed using the statistical package for social sciences (SPSS), version 22 (SPSS Inc., Chicago, IL, USA). Quantitative data are expressed as average ± standard deviation (SD), while qualitative data are expressed as numbers. The Shapiro–Wilk test was used for normality distribution. The distribution of the normality was checked using the independent T-test. In the univariate analysis of the variables in the study, the Kruskal–Wallis and Wilcoxon tests were used according to the variable type and the assumptions. The differences between the categories of the observations in the categorical variables were tested using the chi-squared test Wilcoxon test in the analysis of dependent data, which was applied. A *p*-value of <0.05 was considered statistically significant.

## 3. Results

This study included 79 RFA patients (34 females and 45 males) with a mean age of 55.8 ± 9.6 years and 80 ESWT patients (20 females and 60 males) with a mean age of 49.1 ± 9.5 years. The mean BMI values were 28.52 ± 3.8 in the RFA group and 28.7 ± 2.6 in the ESWT group. Regarding age and BMI, there were no significant differences between the patient groups (*p*: 0.095, *p*: 0.795). The results are shown in Table 1.

There was a significant decrease in VAS scores after treatment in both the RFA and ESWT groups (z: −4.98, z: −5.18, respectively, *p*: 0.00). The decreases in FFI pain, FFI activity restriction, FFI disability, and RM scores were significant in both groups, but the scores after treatment were lower in the RFA group. The before and after treatment measurement values for the patient groups are given in Table 2.

## 4. Discussion

In this study, we compared the effectiveness of ESWT and RFA in the treatment of calcaneal spurs. Although many methods have been suggested for the treatment of CSs, there is still limited evidence. The study investigating the efficacy of ESWT and RFA has not been identified in the literature, so we believe that our study adds to the literature on CS treatment. It was observed that both treatment methods were effective in reducing pain, and although the FFI disability and activity limitation scores of patients in the RFA group were higher, the post-treatment scores were significantly reduced compared to ESWT. The BMI values of our patients (*p*: 0.795), as well as the pre-treatment VAS, FFI pain, and RM scores, were similar in both groups (*p*: 0.83, *p*: 0.22, *p* < 0.01, respectively). Similar improvements in pain scores were observed in both the ESWT and RFA groups.

One of the methods used in the treatment of plantar fasciitis is ESWT, which uses high- or low-energy shock waves to treat the interface between calcaneus and plantar fascia. Although the mechanism of this method in reducing pain is not known precisely, various mechanisms have been proposed. The air gaps developing in the tissues are thought to physically separate the plantar fascia from the calcaneus as a result of ESWT administration, causing transdermal release. It has not been clearly demonstrated that the reduction in pain after ESWT administration may be the result of calcaneal nerve damage and whether this effect is temporary or permanent [26]. Numerous clinical trials have been conducted on ESWT efficacy in plantar fasciitis in patients with calcaneal spur detection. A useful method in symptomatic patients resistant to conservative treatment. Weil et al. found satisfactory results in 82% of patients treated with ESWT [27,28].

Our study showed statistically significant effects of ESWT on pain and FFI scores. Improvements in VAS scores were higher in the ESWT group than RFA. Improvement in VAS scores with ESWT and RFA was parallel with the literature [29,30].

We observed significant improvements in daily life activities in the treatment group with RFA compared to the ESWT group. The improvements in VAS, FFI, and RM scores were significant in the RFA treatment group. Although the activity limitation scores of patients were higher in the RFA group, it was observed that there was a significant decrease in the score after treatment. Our data support the notion that patients who suffer from more restraint in their daily activities may prefer RFA over ESWT. There are studies demonstrating that the reduction in pain with RFA begins in the first month and continues into the 12th month. In patients who underwent RFA treatment, statistically significant improvements were observed in VAS and ankle-heel scoring in evaluations conducted at 1 and 6 months [31]. There are also publications showing that the pain-relieving feature of ESWT varies between 6 and 12 months [32]. This study found significant improvements in VAS and FFI scores in our patients during the sixth month of evaluation. Pain reduction was more significant with ESWT.

An electrode, ultrasound (USG), or fluoroscopy can be used during the procedure. However, successful results have also been reported without the use of USG or fluoroscopy [33]. In this study, we did not use USG or fluoroscopy during the procedure. We repositioned the probes on the heel in foot or foot movements to prevent possible neurological injuries. No complications were seen in any of our patients.

According to our results, RFA is especially in patients with severe pain and limited daily activity, who do not respond to conservative treatment can be a useful alternative to surgery or ESWT. Erken et al. suggested that RFA is an effective alternative treatment option for patients with resistant plantar fasciitis who do not respond to other conservative treatments [34]. Similarly, Landsman et al. [35] showed that RFA is an effective method of treating plantar fasciitis. Despite being invasive, RFA can also prevent certain associated complications, such as local hematoma and neuropathic pain. Local hematoma and neuropathic pain were not seen in our study. RFA is also useful for patients who have failed conservative treatment and ESWT but do not want to risk traditional surgery. It has the advantage of fewer complications and a faster recovery time compared to surgery.

In patients with calcaneal spurs, PF causes pain and difficulty in daily work. ESWT and RFA are preferable methods for patients who do not benefit from conservative methods of treatment. Our study’s strength is that it is the first to evaluate RFA and ESWT in calcaneal spurs. The RFA patient group had higher VAS and FFI scores. Therefore, we could not clarify which method could be more useful. Studies comparing the two methods should be made between groups with the same VAS and FFI scores. Our work is retrospective. We should also say that the follow-up time is short, and different results can be achieved in long-term follow-up.

According to our results, both methods are beneficial, and the choice of treatment should be based on the patient’s complaints and needs. Our results have shown that, according to RFA, ESWT also showed a significant reduction in pain. ESWT, a non-invasive technique, can be selected for patients with pain at the forefront. On the other hand, RFA can be preferred by patients with more physical activity limitations. We think that the improvement in functional scores in RFA is a safe method that is preferable to ESWT in patients with physical activity limitations.

## 5. Conclusions

As a result, ESWT appears more effective in VAS scores, and RFA appears more effective in functional scores.

## Figures and Tables

**Table 1:** Results of patient groups.

	RFA (*n*: 79)	ESWT (*n*: 80)	*p*
**BMI (kg/m^2^)**	28.52 ± 3.8	28.7 ± 2.6	0.795
**Age (year)**	55.8 ± 9.6	49.1 ± 9.5	0.095
**Visual Analog Scale**			
**Before treatment**	7.26	7.34	0.838
**End of treatment**	2.6	1.15	0.025
**FFI pain**			
**Before treatment**	55.43	52.31	0.229
**End of treatment**	2.86	9.05	0.038
**FFI disability**			
**Before treatment**	15.86	27	0.005
**End of treatment**	1.24	4.62	0.02
**FFI activity restriction**			
**Before treatment**	55.76	40.79	<0.001
**End of treatment**	1.8	10.72	0.072

FFI: Foot Function Index; ESWT: Extracorporeal Shock Wave Therapy; RFA: Radiofrequency Ablation; BMI; Body Mass Index.

**Table 2:** Patient groups before and after measurement values.

	RFA Min–Max (Median)	ESWT Min–Max (Median)
Visual Analog Scale		
Before treatment	2–10 (7.26)	0–10 (7.3)
End of treatment	0–10 (2.6)	0–8 (1.15)
Change according to BT p	0.00 (z: −4.98)	0.00 (z: −5.18)
FFI pain		
Before treatment	36–81 (55.4)	20–76 (52.31)
End of treatment	0–40 (2.8)	0–60 (9.05)
Change according to BT p	0.00 (z: −5.64)	0.00 (z: −5.16)
FFI disability		
Before treatment	2–69 (15.86)	0–81(27)
End of treatment	0–20 (1.24)	0–27(4.62)
Change according to BT p	0.00 (z: −5.58)	0.00 (z: −5.12)
FFI activity restriction		
Before treatment	21–79 (55.76)	0–76(40.79)
End of treatment	0–40 (1.8)	0–59 (10.72)
Change according to BT p	0.00 (z: −5.64)	0.00 (z: −5.14)
RM		
Before treatment	3–4 (3.64)	2–4 (3.34)
End of treatment	1–2(1.05)	1–3(1.29)
Change according to BT p	0.00 (z: −5.84)	0.00 (z: −5.14)

VAS: Visual Analog Scale; RM: The Roles and Maudsley score; FFI: Foot Function Index; ESWT: Extracorporeal Shock Wave Therapy; RFA: Radiofrequency Ablation; BT: Before treatment.

## Data Availability

The accession numbers will be provided during the review.
